# Reduced Responses of Submucous Neurons from Irritable Bowel Syndrome Patients to a Cocktail Containing Histamine, Serotonin, TNFα, and Tryptase (IBS-Cocktail)

**DOI:** 10.3389/fnins.2015.00465

**Published:** 2015-12-16

**Authors:** Daniela Ostertag, Sabine Buhner, Klaus Michel, Christian Pehl, Manfred Kurjak, Manuela Götzberger, Ewert Schulte-Frohlinde, Thomas Frieling, Paul Enck, Josef Phillip, Michael Schemann

**Affiliations:** ^1^Human Biology, Technische Universität MünchenFreising, Germany; ^2^Academic Hospital VilsbiburgVilsbiburg, Germany; ^3^Medical Center for GastroenterologyMünchen, Germany; ^4^Academic Hospital FreisingFreising, Germany; ^5^Helios Clinic KrefeldKrefeld, Germany; ^6^Academic Hospital TübingenTübingen, Germany

**Keywords:** irritable bowel syndrome, enteric nerve desensitization, immune mediators, colonic biopsy, submucous plexus, calcium imaging

## Abstract

**Background and Aims:** Malfunctions of enteric neurons are believed to play an important role in the pathophysiology of irritable bowel syndrome (IBS). Our aim was to investigate whether neuronal activity in biopsies from IBS patients is altered in comparison to healthy controls (HC).

**Methods:** Activity of human submucous neurons in response to electrical nerve stimulation and local application of nicotine or a mixture of histamine, serotonin, tryptase, and TNF-α (IBS-cocktail) was recorded in biopsies from 17 HC and 35 IBS patients with the calcium-sensitive-dye Fluo-4 AM. The concentrations of the mediators resembeled those found in biopsy supernatants or blood. Neuronal activity in guinea-pig submucous neurons was studied with the voltage-sensitive-dye di-8-ANEPPS.

**Results:** Activity in submucous ganglia in response to nicotine or electrical nerve stimulation was not different between HC and IBS patients (*P* = 0.097 or *P* = 0.448). However, the neuronal response after application of the IBS-cocktail was significantly decreased (*P* = 0.039) independent of whether diarrhea (*n* = 12), constipation (*n* = 5) or bloating (*n* = 5) was the predominant symptom. In agreement with this we found that responses of submucous ganglia conditioned by overnight incubation with IBS mucosal biopsy supernatant to spritz application of this supernatant was significantly reduced (*P* = 0.019) when compared to incubation with HC supernatant.

**Conclusion:** We demonstrated for the first time reduced neuronal responses in mucosal IBS biopsies to an IBS mediator cocktail. While excitability to classical stimuli of enteric neurons was comparable to HC, the activation by the IBS-cocktail was decreased. This was very likely due to desensitization to mediators constantly released by mucosal and immune cells in the gut wall of IBS patients.

## Introduction

The prevalence of irritable bowel syndrome (IBS) is estimated to be around 10–20% throughout the world. The main symptoms of this functional disease are abdominal pain, bloating, and stool irregularities (Longstreth et al., 2006). Based on whether diarrhea, constipation or a mixture of both prevail, patients may, according to Rome III criteria, be subgrouped as IBS-D, IBS-C, or IBS-M, respectively (Drossman, 2006). The German guidelines define IBS-bloating as another subgroup, in particular if these patients do not report stool irregularities but merely abdominal pain (Layer et al., 2011). So far satisfactory treatment is rarely achieved.

Several mechanisms appeared to be important in the generation of IBS symptoms including motility disorders, altered visceral sensitivity and pain processing, increased mucosal permeability, altered microbiota, and low grade inflammation (see Corsetti et al., 2014). There is strong evidence that the immune system (Bashashati et al., 2014) and its interaction with the enteric nervous system (ENS) is involved in the pathophysiology of IBS. The human ENS consists of the myenteric plexus located between the two outer muscle layers and the submucous plexus with three separate plexuses between the circular muscle and the epithelial layer. The submucous plexus controls transport across the mucosa, microcirculation as well as circular muscle activity (see Schemann and Neunlist, 2004). Immune cells such as mast cells are particularly numerous in the submucous plexus. The anatomical and functional interactions between immune cells, in particular mast cells, and submucous neurons is the basis for neuroimmune interactions in the lamina propria (see Buhner and Schemann, 2012; Schemann and Camilleri, 2013). IBS patients show an increased number of mast cells in close proximity to submucous neurons (Barbara et al., 2004; Balestra et al., 2012). The mast cell to enteric nerve signaling is primarily through the release of histamine and tryptase, which are known to activate neurons of the submucous plexus (Breunig et al., 2007; Mueller et al., 2011). Accordingly, a supernatant obtained from stimulated mast cells had an excitatory effect on guinea pig and human enteric ganglia (Schemann et al., 2005). Furthermore, brief application (several 100 ms) of mucosal biopsy supernatants of IBS patients but not of healthy controls activated enteric neurons and this activation involved histamine and proteases (Buhner et al., 2009). The relevance of altered neuroimmune interactions in IBS is supported by the finding that a panel of chemokines and cytokines as well as antibodies and factors involved in wound healing and mucosal regeneration were upregulated in the serum of IBS patients. The role of blood borne factors was demonstrated by the sensitizing effect of supernatants of peripheral blood mononuclear cells (PBMC) of IBS-D patients on mouse colonic afferent nerve endings. This effect was mainly due to the increased concentration of TNF-α (Hughes et al., 2013). Apart from immune cells, there are also enteroendocrine cells in the epithelium that modulate nerve activity by release of their mediators. One important mediator is serotonin, which is enhanced in IBS (Crowell, 2004). Although not significantly increased in mucosal biopsy supernatants of patients with IBS (Coates et al., 2004), serotonin contained in mucosal biopsy supernatants contributes to neuronal activation (Buhner et al., 2012) as well as to the symptoms of IBS patients (Gershon, 2005).

These and other findings strongly suggest that IBS patients may experience an altered activation profile of enteric neurons. So far studies have been using biopsies of IBS patients to study mediator release, nerve, and mast cell density or receptor expression (Nasser et al., 2014). Our aim was to directly measure neuronal activation in tissue samples of the submucous plexus of IBS patients to study altered neuronal behavior in IBS. The stimuli used to challenge enteric neurons were: electrical stimulation of neural synapses, activation of nicotinic receptors reflecting the action of the most important excitatory transmitter acetylcholine and a mediator cocktail mimicking an IBS-like milieu.

## Materials and methods

### Study participants and tissue samples

We originally recruited 190 healthy volunteers and 106 IBS patients. We obtained suitable biopsies from 17 healthy volunteers (58 ± 12 years; 7 female and 10 male) and 35 patients with IBS (48 ± 13 years; 26 female and 9 male); for details see Results. All IBS patients were subtyped according to predominant stool irregularities following the ROME III consensus as IBS-D (16 patients; 46 ± 14 years; 11 female and 5 male), IBS-C (8 patients; 51 ± 14 years; 6 female and 2 male), or IBS-M (5 patients; 47 ± 7 years; 4 female and 1 male). Following the German guidelines which do not exclusively consider stool patterns, six additional patients were subtyped as IBS-bloating with abdominal pain but no stool irregularities (50 ± 11 years; 5 female and 1 male). Details of patients and healthy volunteers are found in Table 1. As outlined in Table 1 some patients took medication but all patients had symptoms at the time of endoscopy. Endoscopies were performed in primary or secondary care centers for gastroenterology in Munich, Freising, or Vilsbiburg, respectively. Healthy volunteers were undergoing routine endoscopy for cancer screening. All volunteers were asked to fill out the German equivalent of the self-report Rome III Modular Questionnaire for IBS (Drossman, 2006). Exclusion criteria for healthy controls were symptoms related to the GI tract. This was either given if healthy volunteers matched positive for ROME III criteria or reported one or more of the following symptoms: (a) abdominal pain for 1 day per month or more (for women: not related to menses) (b) hard or lumpy stool more than 25% of the time (c) loose, mushy or watery stool more than 25% of the time. Exclusion criterion for both healthy controls and IBS patients was prior surgery of the gastrointestinal tract. All IBS patients presented themselves to the medical institutions after experiencing gastrointestinal symptoms. Patients were diagnosed by experienced gastroenterologists according to Rome III criteria and the German guidelines (for IBS-bloating). The procedure of taking biopsies was similar to the method already described by Cirillo et al. (2013). Four biopsies were taken from the sigmoid colon with standard biopsy forceps without needle (Ø 2.3 mm; Pauldrach, Garbsen, Germany). After removal all biopsies were immediately transferred to a sterile glass bottle containing 10 ml of ice-cold, sterile Hepes/Krebs solution (in mmol/l: 135 NaCl, 3 Hepes, 12.2 Glucose, 5.4 KCl, 1 MgCl_2_.6H_2_O, 1.25 CaCl_2_.2H_2_O, 1.2 NaH_2_PO_4_; pH = 7.4) and transported to the laboratory. The duration of the transport lasted between 20 min and 2 h during which the samples were kept at 4°C. All procedures were approved by the ethics committee of the Technische Universität München (2595/09). Written confirmed consent was obtained from all participants.

**Table 1:** **Characteristics of IBS patients and healthy volunteers**.

**Clinic**	**Age**	**Gender**	**Medication**	**Diagnosis**	**Clinic**	**Age**	**Gender**	**Diagnosis**
Munich	39	f	none	IBS-Bloating	Freising	27	f	Healthy Control
Munich	39	f	none	IBS-Bloating	Freising	32	f	Healthy Control
Munich	49	m	none	IBS-Bloating	Freising	62	f	Healthy Control
Vilsbiburg	62	f	none	IBS-Bloating	Freising	63	f	Healthy Control
Vilsbiburg	68	f	none	IBS-Bloating	Freising	47	m	Healthy Control
Freising	48	f	none	IBS-C	Freising	56	m	Healthy Control
Munich	43	f	none	IBS-C	Freising	59	m	Healthy Control
Munich	71	f	none	IBS-C	Freising	60	m	Healthy Control
Munich	53	m	none	IBS-C	Freising	64	m	Healthy Control
Vilsbiburg	27	f	Laxative	IBS-C	Freising	69	m	Healthy Control
Vilsbiburg	40	f	none	IBS-C	Freising	76	m	Healthy Control
Vilsbiburg	43	f	none	IBS-C	Munich	58	f	Healthy Control
Vilsbiburg	53	f	Bisacodyl	IBS-C	Munich	59	f	Healthy Control
Vilsbiburg	71	f	none	IBS-C	Munich	63	f	Healthy Control
Munich	39	f	none	IBS-D	Munich	58	m	Healthy Control
Munich	42	f	none	IBS-D	Vilsbiburg	58	m	Healthy Control
Munich	45	f	none	IBS-D	Vilsbiburg	67	m	Healthy Control
Munich	47	f	none	IBS-D				
Munich	54	f	none	IBS-D				
Munich	67	f	none	IBS-D				
Munich	67	f	none	IBS-D				
Munich	31	m	none	IBS-D				
Munich	32	m	Loperamid	IBS-D				
Munich	37	m	none	IBS-D				
Munich	48	m	none	IBS-D				
Munich	56	m	none	IBS-D				
Vilsbiburg	16	f	Butylscopolamin	IBS-D				
Vilsbiburg	23	f	none	IBS-D				
Vilsbiburg	45	f	Mebeverin	IBS-D				
Vilsbiburg	54	m	none	IBS-D				
Munich	41	f	none	IBS-M				
Munich	47	f	none	IBS-M				
Munich	52	f	none	IBS-M				
Vilsbiburg	36	f	none	IBS-M				
Vilsbiburg	57	m	Probiotics	IBS-M				

Submucosal tissue from three male guinea-pigs (Dunkin Hartley, Harlan Winkelmann, Borchen, Germany) was used for over-night incubation with mucosal biopsy supernatants. The animals were sacrificed by cervical dislocation and exsanguination in accordance to German guidelines for animal welfare and protection (Deutsches Tierschutzgesetz) and approved by the Bavarian state ethics committee (Regierung Oberbayern, which serves as the Institutional Care and Use Committee for the Technische Universität München) according to §11 Deutsches Tierschutzgesetz under the reference number 32-568. Colonic segments were removed immediately.

### Tissue preparation

Immediately after arrival the human tissue samples were prepared for the imaging experiments in ice-cold, oxygenated Krebs solution for dissection (in mmol/l: 117 NaCl, 25 NaHCO_3_, 11 Glucose, 4.7 KCl, 1.2 MgCl_2_.6H_2_O, 2.5 CaCl_2_.2H_2_O, 1.2 NaH_2_PO_4_; pH = 7.4, 293 mosmol/l). To maintain the physiological milieu and to mimic *in vivo* conditions as closely as possible, no further dissection was performed and therefore the mucosa remained a part of the preparation. For the imaging experiment the whole, undissected biopsy was gently stretched and pinned flat on a Sylgard-platelet with the submucous ganglion layer facing upwards. Connective tissue was removed so that the inner submucous plexus was accessible.

For the over-night incubation of guinea pig submucous plexus preparations a 5 × 5 mm piece of the distal colon was used. The muscle layers and the mucosa were removed and the submucous plexus was stretched and pinned flat on a Sylgard-platelet. After dissection, the Sylgard-platelet with the submucous plexus preparation was transferred to a petri-dish. Under sterile conditions an incubation chamber made of Sylgard with an inner opening of 5 × 5 mm was mounted on top of the Sylgard-platelet. We verified in preliminary experiments that the solutions inside and outside of the chamber were completely separated from each other (data not shown). Two preparations of submucous plexus of the same guinea pig were incubated in parallel. Supernatants were collected as previously published (Barbara et al., 2007). The preparation was incubated with 100 μl of a 1:10 dilution of biopsy supernatants from either an IBS patient (IBS-D 11, female) or healthy control (HC 07, female) for 16–18 h at 37°C with 5% CO_2_. To guarantee a moisturized atmosphere and to prevent evaporation, the petri-dish was filled with 3 ml of Krebs solution that surrounded the incubation chamber.

### Imaging method

The previously described fast imaging technique (Schemann et al., 2002) was used with small modifications. To facilitate the recording of neuronal activity in human biopsies with attached mucosa, all experiments were performed with an upright Zeiss Examiner D1 microscope with water immersion objective (Plan-Apochromat 40x/1.0 DIC M27, Zeiss, Oberkochen, Germany). The same settings were used for experiments with guinea-pig submucous plexus preparations.

To record neuronal activity in human colonic biopsies calcium-imaging with 10 μmol/l Fluo-4 AM (Invitrogen, Darmstadt, Germany) was used to monitor intracellular calcium increase ([Ca^2+^]_i_) (Michel et al., 2011). As previously described, ganglia and their neuronal cell bodies including their shape were easily detected in the bright field image as well as during the rise of [Ca^2+^]_i_ (increased Fluo-4 fluorescence), respectively (Michel et al., 2011; Mueller et al., 2011). *Post-hoc* immunohistochemistry was performed in some preparations using PGP 9.5 as previously described (Mueller et al., 2011) to verify identification of submucous neurons. Tissue preparations mounted on the Sylgard-platelet were incubated for 45 min in Fluo-4 AM containing Krebs buffer and subsequently washed for 20 min. The Krebs solution (in mmol/l: 117 NaCl, 20 NaHCO3, 11 Glucose, 4.7 KCl, 1.2 MgCl2.6H2O, 2.5 CaCl2.2H2O, 1.2 NaH2PO4; pH = 7.4; 293 mosmol/l) used for incubation, washing and perfusion during the experiment contained 500 μmol/l Probenecid to prevent dye leakage. Submucous neurons in IBS and HC biopsies were challenged with brief spritz application of 100 μmol/l nicotine, 60 μA electrical stimulation (20 Hz train stimulation) of interganglionic fiber tracts and a mixture containing 1 μmol/l histamine, 1 μmol/l serotonin, 10 nmol/l tryptase (Merck KGaA, Darmstadt, Germany), and 150 pg/ml TNF-α (Biomol GmbH, Hamburg, Germany). At these concentrations the mediators did not evoke any or very weak responses when applied individually (own unpublished results). As previously shown, much higher concentrations were necessary to detect nerve activation by these mediators (Michel et al., 2005; Breunig et al., 2007; Mueller et al., 2011). The concentrations of the mediators were calculated following the mean values measured in IBS supernatants (Buhner et al., 2009) and PBMC supernatants (Hughes et al., 2013) of IBS patients, taking into account that with our microejection system there is a 1:10 dilution before a substance reaches the ganglion (Breunig et al., 2007). The mixture was chosen to mimic some nerve activating components of a mediator cocktail found in colonic biopsy supernatants and serum of IBS-patients (Buhner et al., 2009; Hughes et al., 2013) and will be referred to as IBS-cocktail further on in this paper. The functional relevance relates to the higher levels of the mediators in the samples or the finding that antagonists of the mediators reduced the nerve activation by the biopsy or PBMC supernatants.

To record neuronal activity from guinea-pig submucous neurons the voltage-sensitive dye di-8-ANEPPS (20 μmol/l; Life Technologies, Darmstadt, Germany) was used as described previously (Neunlist et al., 1999). Ganglia of the IBS-conditioned and the HC-conditioned preparations were challenged with short term spritz application of 100 μmol/l nicotine and the IBS-supernatant (IBS-D 11) that had been used for the incubation.

All experiments were performed at 37°C and all reagents were purchased from Sigma-Aldrich, if not stated otherwise.

### Data analysis and presentation

Analysis of imaging experiments was performed with Neuroplex 10.1.2. (*RedShirtImaging, Decatur, GA, USA*). For the calcium-imaging experiments the maximal intracellular calcium increase relative to resting light level (ΔF/F) was determined for each application. As the staining with Fluo-4 AM does not outline single cells in the ganglion, it is not possible to directly deduce the number of neurons per ganglion, the percentage of responding area in each ganglion was calculated instead. As previously described, the maximal calcium increase, and the percentage of responding area per ganglion were multiplied to obtain the neuroindex, a parameter best reflecting the overall neuronal activity level (Buhner et al., 2014). Throughout the manuscript the neuroindex values are presented without units. For the experiments with di-8-ANEPPS action potentials were counted per neuron and the number of neurons responding per ganglion was deduced from the image. The spike frequency was multiplied with the percentage of responding neurons to again obtain a neuroindex (Buhner et al., 2014).

All statistics and graphs were performed with Sigma Plot 12.5 (*Systat Software Inc., Erkrath, Germany*). Data in the figures are presented as box-and-whisker plots illustrating the median with 25th and 75th quartiles and 10th and 90th percentiles. For statistical analysis the Mann-Whitney Rank Sum Test, ANOVA on ranks with Dunn's method as *post-hoc* test, Spearman Rank Order and Fisher exact test were implemented. To compare the age of the study participants, the Student *t*-test was used and data are given as mean ± SD. Differences were considered significantly different when *P* ≤ 0.05.

## Results

Contrary to previous work (Lebouvier et al., 2010) it was not common that the routine colonic biopsies, as taken in primary and secondary care centers and as approved by the ethical committee of our University, contained sufficient viable submucous ganglia to perform calcium imaging. Originally we recruited 190 HC and 106 IBS patients for this study. Of the HC biopsies, 79 and 67 did not contain enough submucous plexus layer or no viable ganglia, respectively. Of the remaining 44 controls, 27 had to be excluded because seven reported non-IBS related gut symptoms at the time of endoscopy, 19 did not provide enough information to include them as healthy controls and one turned out to be IBS-C. Of the biopsies from 106 IBS patients, 41 and 29 did not contain enough submucous plexus layer or no viable ganglia, respectively. Of the remaining 36 patients with at least one viable ganglion in one of the four biopsies, two had to be excluded because the diagnosis IBS was not according to the guidelines used in this study. In total 17 confirmed healthy controls and 29 confirmed IBS patients fulfilling ROME III criteria (Drossman, 2006) as well as six confirmed IBS patients fulfilling criteria established by the German Society for Metabolic and Digestive Diseases (Layer et al., 2011) were eligible to be included. According to the German guidelines diagnosis of IBS also applies to patients with bothersome bloating but without stool irregularities. This group is referred to as IBS-bloating.

We recorded from a sufficient number of ganglia to analyze responses to electrical stimulation, nicotine, and IBS-cocktail in biopsies from IBS-C, IBS-D, and IBS-bloating. It was not possible to record responses to all stimuli in biopsies from IBS-M patients.

### Neuronal activity after nicotine application was not altered in biopsies from IBS patients

**Figure 1:**
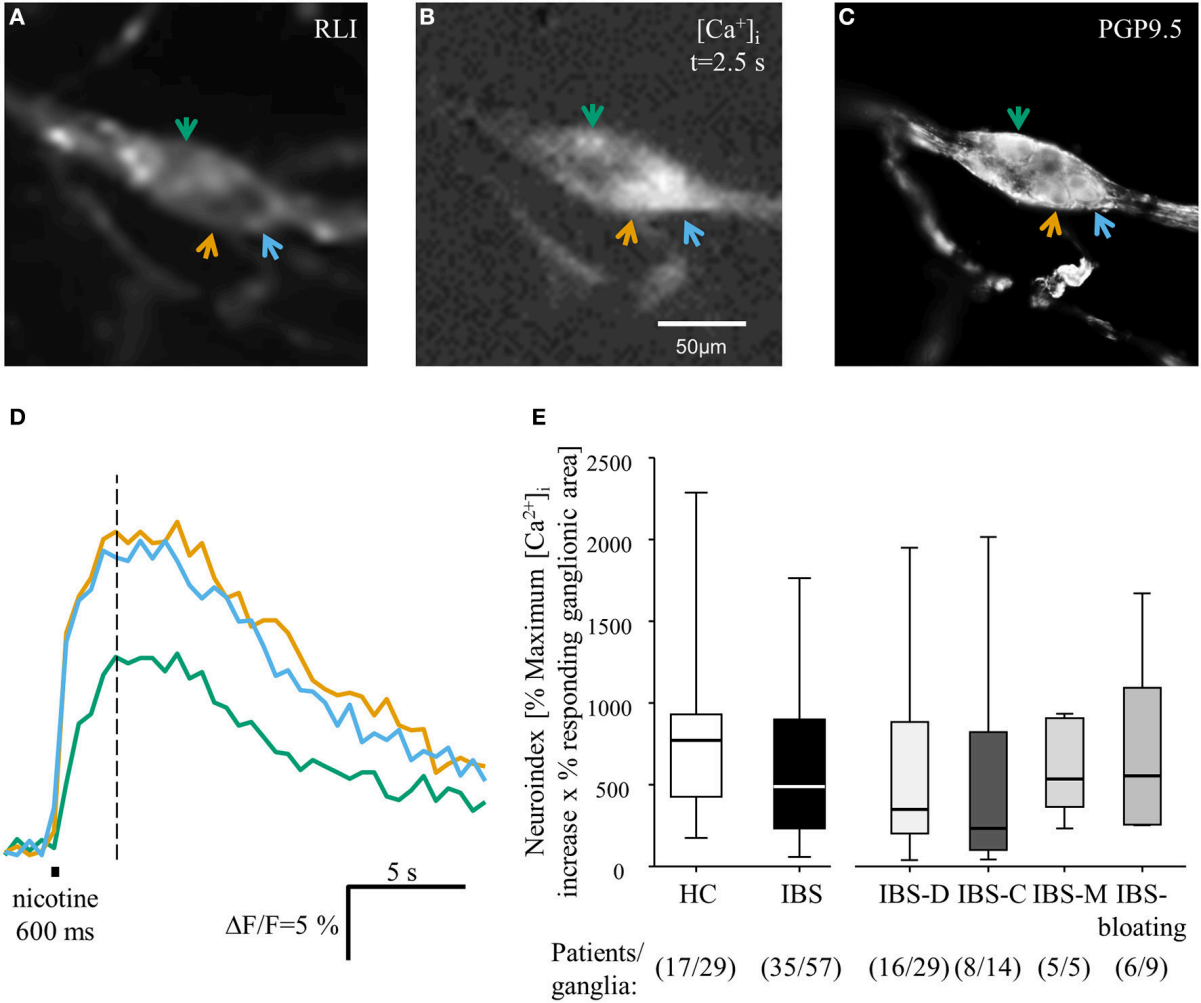
**Neuronal responses to the spritz application of 100 μmol/l nicotine in human submucous ganglia obtained from IBS and HC biopsies**. **(A)** Image of a Fluo-4 AM labeled ganglion before nicotine application taken with the low resolution CCD camera. **(B)** Image 2.5 s after nicotine application showing a peak response in three large diameter cells marked by arrows. **(C)** The same ganglion after PGP 9.5 staining revealed that the three responding cells were neurons (image taken with a high resolution camera of the fluorescent microscope). **(D)** Trace of the three neurons reflect the intracellular calcium increase in response to the application of nicotine (colored traces respond to colored arrows in **A–C**). **(E)** Nicotine evoked responses did not differ between HC and IBS ganglia. The increase in the neuroindex was comparable. There were also no differences among IBS subgroups.

HC and IBS biopsies were challenged with brief spritz application of nicotine. Nicotine was chosen as a general neural activator because most enteric neurons express functional nicotinic receptors (Schemann et al., 2002). Nicotine evoked an increase in [Ca^2+^]_I_ in 97% of ganglia in HC (28 out of 29 ganglia, 17 patients) and in 93% of ganglia in IBS biopsies (53 out of 57 ganglia, 35 patients; *P* = 0.659). Maximal [Ca^2+^]_I_, the percentage of responding ganglionic area and the neuroindex evoked by nicotine were not different between HC and IBS ganglia (*P* = 0.402, *P* = 0.185, and *P* = 0.097, respectively). There was also no difference in the nicotine responsiveness between ganglia from the four different IBS subgroups ([Ca^2+^]_I_: *P* = 0.698; percentage responding ganglionic area: *P* = 0.643; neuroindex: *P* = 0.580) (Figure 1).

In the German health system, colon cancer screening is paid for by the insurance companies starting at the age of 55. This was the main age group recruited for the healthy control cohort. This meant that our healthy participants were slightly yet still significantly older than the IBS patients (*p* = 0.014). It is therefore important however to stress that the nicotine evoked neuroindex was independent of age (*P* = 0.302; correlation coefficient *r*: −0.262). There was also a significant difference for the ratio of men and women in both groups (*P* = 0.032). However, there was no difference between the nicotine evoked neuroindex between male and female subjects (*P* = 0.438).

We were concerned that IBS biopsies may be more sensitive to damage than those from HC. Evidence for this would be that nicotine activated a smaller ganglionic area. This was not the case as the absolute ganglionic area responding to nicotine was 1500 μm^2^ in IBS and 2000 μm^2^ in HC ganglia (*P* = 0.76).

### Responses to electrical stimulation of interganglionic fiber tracts was not altered in biopsies from IBS patients

**Figure 2:**
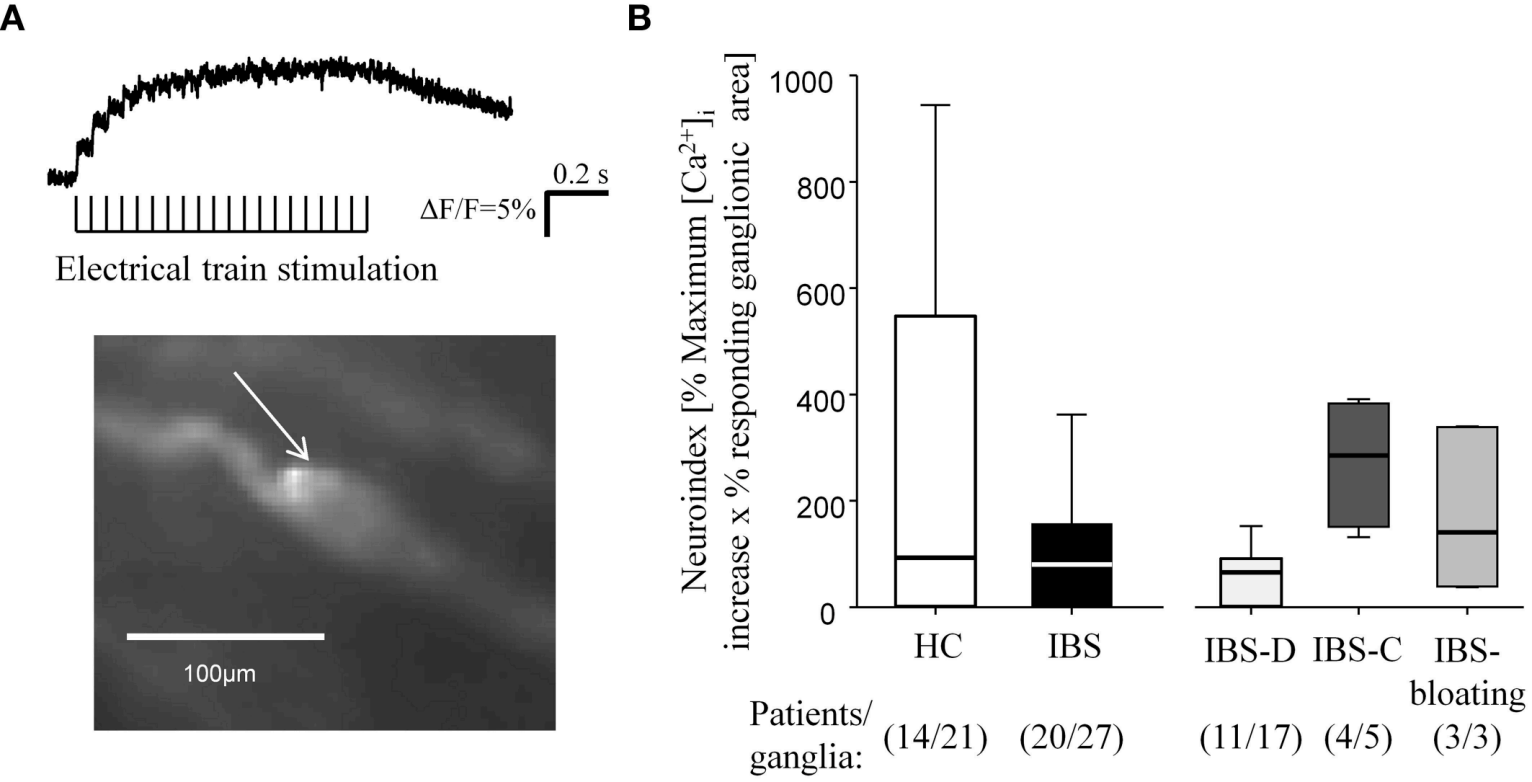
**Neuronal response to electrical stimulation of interganglionic fiber tracts**. **(A)** Submucous ganglion stained with the calcium-sensitive dye Fluo-4 AM. The trace reflects [Ca^2+^]_i_ transients in response to electrical stimulation in the cell marked by the white arrow. **(B)** Electrical stimulation evoked comparable increases in the neuroindex in IBS and HC biopsies as well as between IBS subgroups.

Electrical stimulation of interganglionic fiber tracts in HC and IBS biopsies evoked activation in a comparable proportion of ganglia (61% in HC and 62% in IBS, *P* = 1; *n* = 14 patients/21 ganglia and 20 patients/27 ganglia, respectively). The neuronal activation was not significantly different between the two groups ([Ca^2+^]_I_: *P* = 0.685; percentage responding ganglionic area: *P* = 0.514; neuroindex: *P* = 0.448) (Figure 2).

### Ganglia of IBS patients responded less to the IBS-cocktail compared to healthy controls

**Figure 3:**
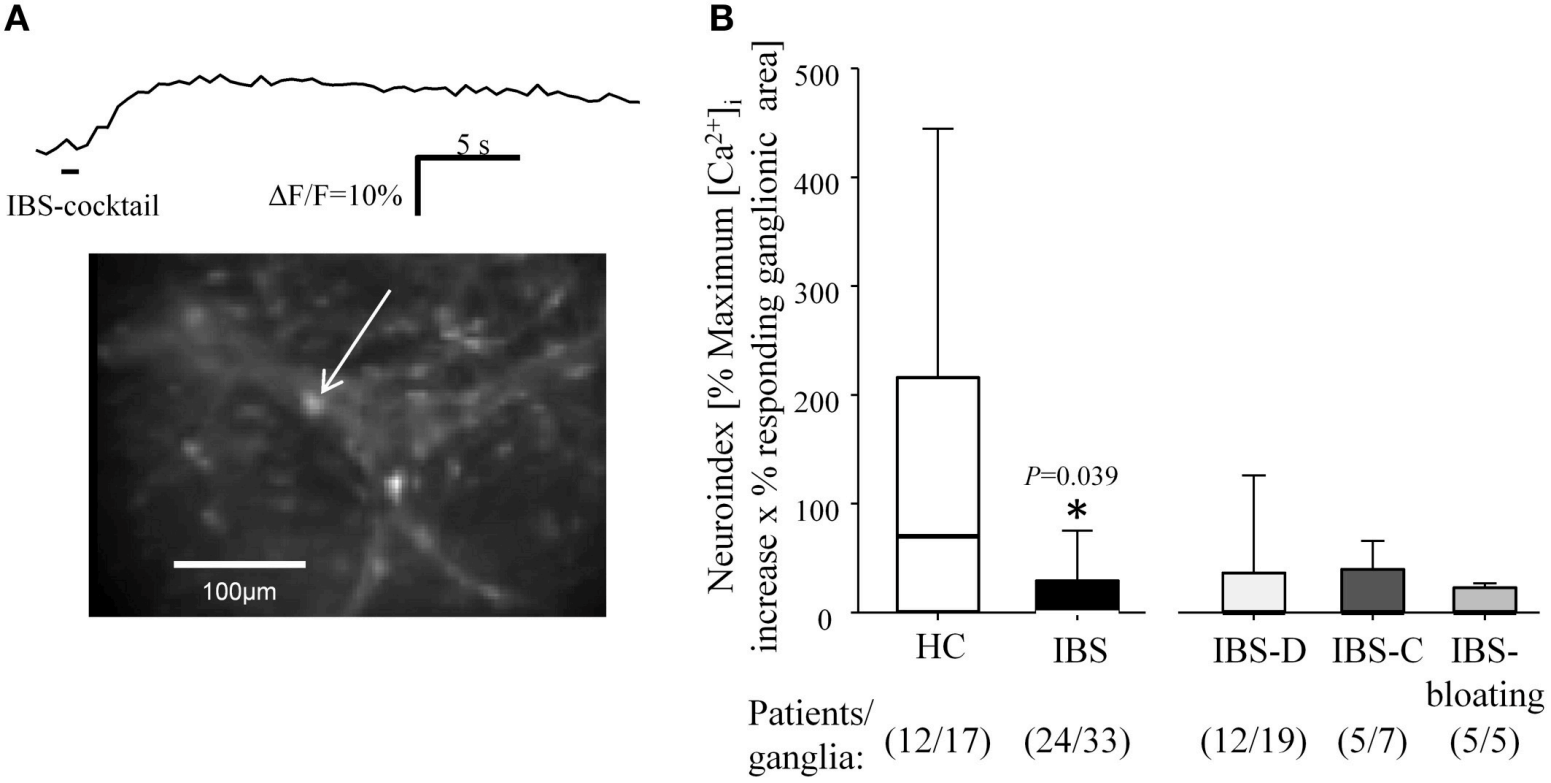
**Neuronal response to the spritz application of an IBS-cocktail on submucous ganglia in IBS and HC biopsies**. **(A)** Submucous ganglion stained with the calcium-sensitive dye Fluo-4 AM. The trace reflects [Ca^2+^]_i_ increase in response to application of the IBS-cocktail in the cell marked by the white arrow. **(B)** The IBS-cocktail evoked significantly smaller neuronal activation in ganglia from IBS biopsies as indicated by the reduced neuroindex (^*^p < 0.05). There was no significant difference between the subtypes IBS-D, IBS-C, and IBS-bloating.

The IBS-cocktail containing serotonin, histamine, tryptase, and TNF-α evoked a [Ca^2+^]_I_ increase in 53% and 30% of ganglia in biopsies from HC (*n* = 12 patients/17 ganglia) and IBS (*n* = 24 patients/33 ganglia), respectively. There was no difference in the proportion of responsive ganglia (*P* = 0.137). The neuroindex in response to application of the IBS-cocktail was significantly lower in submucous ganglia from IBS biopsies compared to HC (*P* = 0.039). This was due to a lower median increase in [Ca^2+^]_I_ (*P* = 0.065) and a decreased responding ganglionic area (*P* = 0.055). The smaller increase in [Ca^2+^]_I_ (*P* = 0.909), the reduced percentage of responding ganglionic area (*P* = 0.962) and the lower neuroindex (*P* = 0.980) were comparable among IBS subgroups. The responding cells were neurons because all exhibited an immediate response to nicotine and/or were PGP 9.5 positive (not always tested, see also Figure 1). In addition, the cell morphology that was revealed during the rise in [Ca^2+^]_I_ signal clearly distinguished small glia cells from large neurons (Mueller et al., 2011). Responses in IBS-M biopsies were not tested because successful recording was only possible in two patients (Figure 3).

We found a significant correlation between the age of an IBS patient and the neuroindex evoked by the IBS-cocktail (*P* = 0.006, *r*: 0.448) suggesting that younger patients responded less to the IBS-cocktail. This was not the case for young healthy controls. We have no explanation for this observation in particular since it was independent of patient gender, IBS subtype or the clinic providing the biopsies, and there was no correlation between the nicotine evoked neuroindex and patient age (*P* = 0.721, *r*: −0.062).

### Over-night conditioning of guinea-pig submucous plexus preparations caused desensitization to IBS mucosal biopsy supernatant

**Figure 4:**
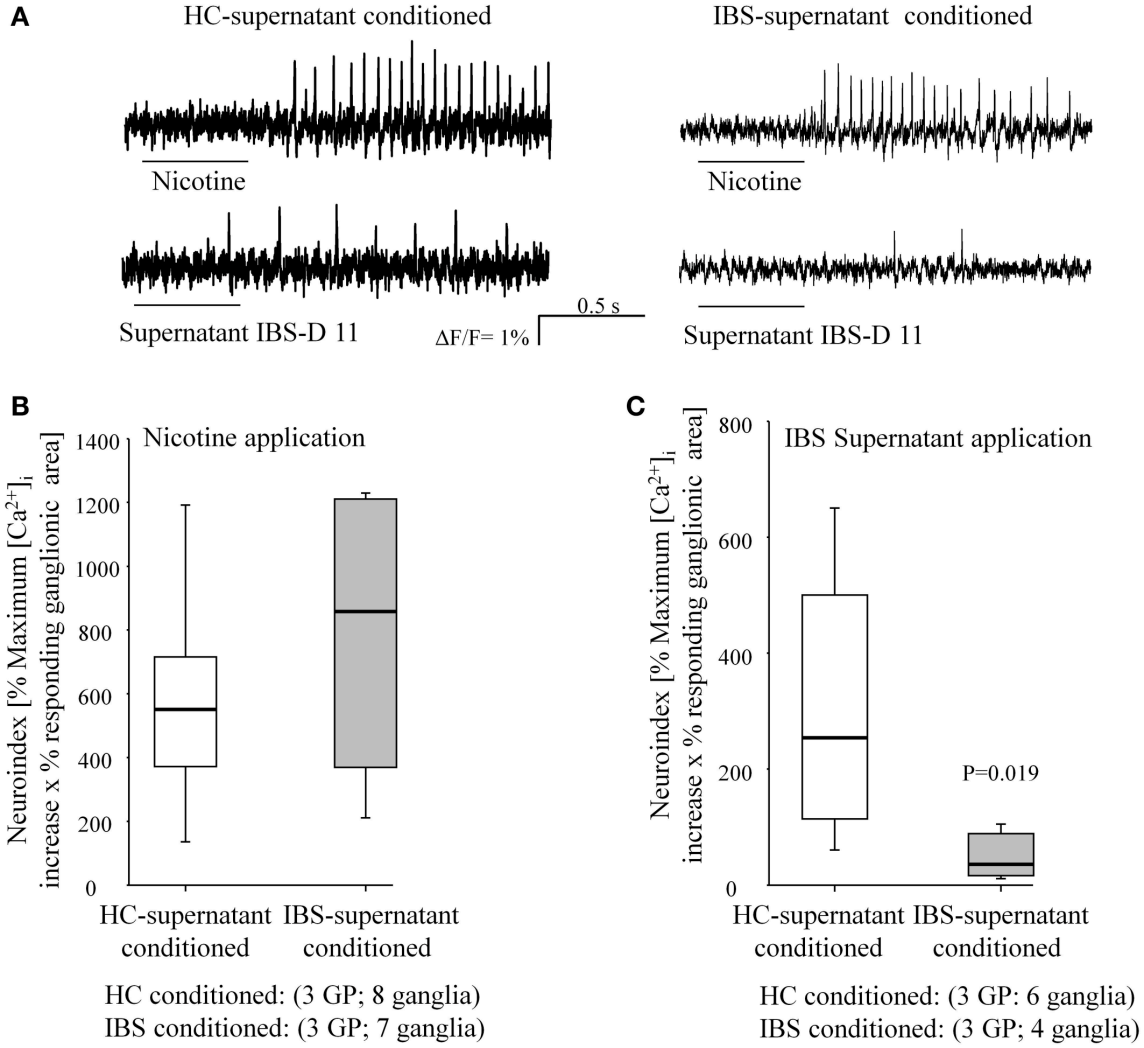
**Overnight incubation with an IBS biopsy supernatant but not with a HC biopsy supernatant desensitized the neurons to application of IBS supernatant**. **(A)** Representative traces to spritz application of nicotine or IBS supernatant in HC and IBS preincubated tissues. **(B)** Nicotine evoked responses, expressed as neuroindex, were comparable in tissues conditioned by IBS or HC supernatants. **(C)** Application of the IBS supernatant which had been used to condition the tissues (= IBS conditioned) evoked a significantly smaller neuronal activity in IBS compared to HC-conditioned tissue as revealed by a reduced neuroindex.

The reduced response to the mediator cocktail in IBS biopsies contrasted with the excitatory action of brief application of IBS mucosal biopsy supernatants which contained the most relevant nerve activating substances (Buhner et al., 2009). Repeated applications of the supernatants by brief microejection (400 ms) every 20 min resulted in reproducible responses in human and guinea-pig enteric neurons (Buhner et al., 2009). We hypothesized that the reduced sensitivity to the IBS-cocktail in IBS-biopsies was the result of long term exposure of neurons and desensitization to the mediators. To test this idea we conditioned guinea pig submucous plexus preparations by overnight incubation with mucosal biopsy supernatants from an IBS patient or a healthy control. The application of nicotine evoked action potential discharge in 100% of the ganglia (*n* = 8) conditioned with the HC supernatant and 100% of ganglia (*n* = 7) conditioned with the IBS supernatant. We only analyzed those ganglia that responded at all to IBS supernatant in order to reveal gradual changes in the responsiveness between HC and IBS supernatant conditioned tissues. The nicotine evoked neuroindex was comparable (*P* = 0.397), as were the spike frequency (*P* = 0.536) and the percentage of responding neurons per ganglion (*P* = 0.463). We then challenged the ganglia with the same IBS supernatant that had been used for conditioning the tissue. The neuroindex evoked by the IBS supernatant in the tissue that had been preincubated with this supernatant was significantly smaller compared to the one in HC preincubated ganglia (*P* = 0.019, Figure 4). This was due to a lower number of spikes (1.8 vs. 4.6 Hz) and to less neurons responding (18 vs. 64% responding neurons).

## Discussion

With this study we have shown for the first time altered neuronal activity in the submucous plexus of IBS patients in response to a combination of mucosal and immune mediators (referred to as IBS-cocktail). Importantly, responses to nicotine and electrical nerve stimulation did not differ between IBS and control biopsies. This is another indication that IBS is not a mere psychosomatic disease but associated with defined organic disorders.

The study revealed a number of novel and also unexpected findings which contrast some current concepts on the pathophysiology of IBS. We and others have shown that short term application of mucosal biopsy supernatants from IBS patients significantly enhanced spike discharge in enteric neurons (Buhner et al., 2009, 2012) and visceral afferents (Hughes et al., 2013) as well as [Ca^2+^]_i_ transients in dorsal root ganglion neurons (Cenac et al., 2007). This led to the hypothesis that nerves in the gut wall of IBS patients are constantly activated and may be even over excitable. It was therefore surprising to find that the response to electrical stimulation of interganglionic fiber tracts or nicotine application did not reveal such enhanced neuronal excitability in IBS biopsies. Both stimuli are well-suited to generally test for neuronal excitability because electrical stimulation enhances synaptic release and nicotine activates the majority of enteric neurons directly via postsynaptic nicotinic receptors. Even more surprising was that the IBS-cocktail that was chosen to mimic enhanced levels of serotonin, histamine, tryptase, and TNF-α found in IBS biopsy supernatants or serum caused a significantly lower neural activation in IBS compared to HC biopsies. Due to the limit in material availability and the fact that the artificial cocktail was much better defined we did not use biopsy supernatants as a stimulus. Although the mediator concentrations vary between biopsy supernatants they were on average higher in IBS compared to healthy controls (Buhner et al., 2009). The concentrations of serotonin, histamine, tryptase, and TNFα were therefore deduced from the concentrations measured in IBS biopsy supernatants (Buhner et al., 2009) or PBMC supernatants (Hughes et al., 2013). There is no reason to believe that lower or supramaximal concentrations may lead to different findings and there is a more likely explanation for the reduced response of the IBS-cocktail in IBS biopsies. We suggest that submucous neurons of IBS patients are constantly exposed to a plethora of mediators among which are those used as a stimulus in our study. In some studies, brief exposure of nerves to such a milieu, mimicked by the IBS biopsy supernatants, immediately, and directly activated them in preparations from healthy human controls or in animal tissue (Barbara et al., 2007; Buhner et al., 2009, 2012, 2014; Hughes et al., 2013). This activation was also observed after individual application of serotonin (Michel et al., 2005), histamine (Breunig et al., 2007), tryptase (Mueller et al., 2011) or TNF-α (O'Malley et al., 2011) to enteric neurons, though much higher concentrations were used in these experiments. The same studies also reported strong receptor desensitization to repeated and/or long term exposure to each of the substances when applied at high concentrations. Apparently, this is what happened in the IBS biopsies. This notion is supported in our study since overnight incubation of submucous plexus preparations with IBS biopsy supernatant caused a substantially decreased responsiveness to the application of the supernatant when compared to its response when incubated with HC biopsy supernatants. For this particular experiment we used the biopsy supernatants in order to mimic as closely as possible the milieu enteric neurons would be exposed to in a healthy or IBS gut. We did not use the IBS-cocktail because we wanted to find out whether we were able to mimic the situation in patients and indeed find desensitization to the IBS but not HC supernatants. The desensitization may be a physiological response to protect nerves from over-excitation by the enhanced release of epithelial and immune mediators in IBS.

Although the above is a plausible interpretation of our findings we have to acknowledge certain limitations of our study. We cannot fully rule out that enteric neurons in biopsies undergo some plastic changes during either the pretreatment of the patients before endoscopy, the handling of the biopsies, the transport, or the tissue preparation for [Ca^2+^]_I_ recording. We were very cautious to keep these influences to a minimum. For example, we used the fastest possible way to transport the biopsies to the laboratory ranging from 20 min to 2 h. In addition we did not perform further dissection of the biopsies which might influence excitability by stressing the tissue. Most importantly however, any particular influence would have affected the IBS biopsies to the same degree as the HC biopsies since all were handled similarly. Since we have seen clear differences we may safely conclude that the reduced action of the IBS-cocktail was indeed a genuine reduced responsiveness of enteric neurons in IBS biopsies. Although there is no clinical or experimental evidence, we cannot rule out that IBS biopsies may be more sensitive to the employed experimental procedures and therefore may release more mediators which result in a more pronounced desensitization. In addition, we can only conclude that the desensitization was present at the time of endoscopy. We assume that there is nerve activation at some state of the disease as there would not be any desensitization without prior receptor activation. In this context it is important to again emphasize that all patients had symptoms at the time of endoscopy independent of whether they took medications or not.

Our method to maintain the mucosal milieu of the biopsies by leaving the mucosa attached to the submucous tissue and thereby preventing a re-adaption to the altered conditions during the incubation period and the duration of the experiment, has never been used before. The method proved feasible and the mucosal cells underneath the submucous plexus did not affect the readout of the calcium imaging. The fact that the spritz application by microejection is a very local stimulus contributed to the fact that the mucosa did not disturb the measurements at all as we found no evidence that the applied substances reached the epithelial cells underneath the plexus.

The reduced responsiveness to the IBS-cocktail was evident in all subgroups of IBS patients independent of whether they had diarrhea, constipation, or bloating. This agrees with our previous finding that IBS biopsy supernatants activated enteric neurons through serotonin, histamine, and protease receptors independent of the prevalence of particular symptoms (Buhner et al., 2012). It was therefore expected to see a decrease of responsiveness in all IBS subgroups. At first sight it seems strange that an IBS-cocktail evokes a smaller neuronal response in patients who experience abdominal pain. However, this is not unexpected because visceral hypersensitivity in IBS patients is linked to activation of nociceptive pathways involving dorsal root ganglion neurons rather than enteric neurons which do not project outside the gut and therefore don't transmit pain signals (Buhner et al., 2014). All our patients experienced abdominal pain but we don't know how many actually show visceral hypersensitivity. An evaluation of visceral sensitivity by barostat distension is not possible in a primary or secondary care setting.

Nicotine and electrical stimulation evoked very similar responses in HC and IBS biopsies. This finding suggested that there is no general desensitization but also no sensitization of enteric neurons.

One study demonstrated a sensitizing effect of long term application of mucosal biopsy supernatants from IBS-D but not from IBS-C patients on cultured dorsal root ganglion neurons (Valdez-Morales et al., 2013). The cell bodies of these neurons are not in close proximity to the mucosal layer and might therefore respond differently to this mediator milieu. In addition, the sensitization was observed as a change in basic electrophysiological properties such as rheobase (just threshold for spike discharge) and the number of action potentials at twice the rheobase. It has not been studied how DRG neurons respond to the supernatant that has been used for the overnight incubation. The enhanced sensitization in nociceptive DRG neurons certainly agrees with the clinical manifestation of pain. We have previously reported that the nerve activating potential of muscosal biopsy supernatants was closely related to visceral sensitivity but not at all to stool irregularities (Buhner et al., 2014).

Desensitization was particularly strong for proteases as protease-activated receptor (PAR) activation can only be triggered once for every receptor. After the ligand is cleaved by the protease and bound to the specific site of the receptor, it internalizes and has to be recycled by the cell to become active again (Déry et al., 1998). In IBS patients, not only is the concentration of tissue proteases elevated, but also the activity of fecal proteases (Tooth et al., 2014). These proteolytic activities seem to be present at all time in the gut. Our data now suggest, that this abundance of activating substances leads to a desensitization of submucous ganglia of IBS patients and makes them less responsive to mediators released by immune and mucosal cells while the interneuronal signaling by the main transmitter acetylcholine is perfectly maintained.

In this study we included bloating predominant IBS patients additionally to the subgroups described by the ROME III criteria, which are characterized by their predominant stool consistency. Bloating as well as abdominal pain (Manning et al., 1978) are major symptoms for IBS and therefore should not be left unstudied. We did not find any differences in neuronal activation between IBS-D, IBS-C, IBS-M, and IBS-bloating patients for the stimulus nicotine. For the application of the IBS-cocktail, the number of IBS-C, and IBS-bloating might have been too low to draw a final conclusion. There was, however, no significant difference between the three groups, as they all showed a reduced response to the IBS-cocktail.

Although the present study showed a reduced response to the mediator cocktail, our finding does not preclude sensitization to other transmitters or non-neuronal mediators in IBS biopsies. Further studies will be necessary to investigate the clinical impact and consequences of the desensitization of submucous ganglia of IBS patients to mediators released by immune and mucosal cells for IBS treatment.

## Author contributions

DO: designed the study and performed the experiments as well as analysis and interpretation of data. She drafted the manuscript, approved the final version, and is accountable for all aspects of the work. CP: made the work possible by providing material and medical council. He critically revised the manuscript and approved it for publication. He is accountable for correct patient characterization and material handling. MK: made the work possible by providing material and medical council. He critically revised the manuscript and approved it for publication. He is accountable for correct patient characterization and material handling. MG: made the work possible by providing material and medical council. She critically revised the manuscript and approved it for publication. She is accountable for correct patient characterization and material handling. ES: made the work possible by providing material and medical council. He critically revised the manuscript and approved it for publication. He is accountable for correct patient characterization and material handling. TF: made the work possible by providing material and medical council. He critically revised the manuscript and approved it for publication. He is accountable for correct patient characterization and material handling. PE: made the work possible by providing technical solutions to characterize healthy controls. He critically revised the manuscript and approved it for publication. He is accountable for correct patient characterization. KM: contributed substantially to the analysis and interpretation of data for the work and revised the manuscript critically. He approved the final version to be published and is accountable for all aspects of the work. SB: contributed to the conception of the study. She critically revised the manuscript and approved it for publication. She is accountable for all aspects of the work. JP: made the work possible by providing material and medical council. He critically revised the manuscript and approved it for publication. He is accountable for correct patient characterization. MS: formed the concept and designed the study as well as made the work possible by obtaining funding. He reviewed the manuscript critically for important intellectual content, gave final approval for publication and is accountable for all aspects of the work.

### Conflict of interest statement

The authors declare that the research was conducted in the absence of any commercial or financial relationships that could be construed as a potential conflict of interest.

## Abbreviations

IBS: irritable bowel syndrome
HC: healthy controls
ENS: enteric nervous system
PBMC: peripheral blood mononuclear cells
[Ca^2+^]_i_: intracellular calcium-increase
TNF-α: tumor necrosis factor alpha
PAR: protease-activated receptor.
